# Effects of a Phosphodiesterase inhibitor on the Browning of Adipose Tissue in Mice

**DOI:** 10.3390/biomedicines10081852

**Published:** 2022-08-01

**Authors:** Da Hea Seo, Eugene Shin, Yong-ho Lee, Se-Eun Park, Ki Taek Nam, Jae-woo Kim, Bong-Soo Cha

**Affiliations:** 1 Department of Endocrinology and Metabolism, Inha University School of Medicine, Incheon 22212, Korea; dahea@inha.ac.kr; 2 Graduate School, Yonsei University College of Medicine, Seoul 03722, Korea; yholee@yuhs.ac; 3 Institute of Endocrine Research, Yonsei University College of Medicine, Seoul 03722, Korea; eugene3625@yuhs.ac; 4 Division of Endocrinology and Metabolism, Department of Internal Medicine, Yonsei University College of Medicine, Seoul 03722, Korea; seraph.park@samsung.com; 5 Division of Endocrinology and Metabolism, Department of Internal Medicine, Kangbuk Samsung Hospital, Sungkyunkwan University School of Medicine, Seoul 03181, Korea; 6 Severance Biomedical Science Institute, Yonsei University College of Medicine, Seoul 03722, Korea; kitaek@yuhs.ac; 7 Department of Biochemistry and Molecular Biology, Yonsei University College of Medicine, Seoul 03722, Korea; japol13@yuhs.ac

**Keywords:** cilostazol, white adipose tissue, beige adipose tissue

## Abstract

Cilostazol is a selective inhibitor of phosphodiesterase type 3 (PDE3) that increases intracellular cyclic adenosine monophosphate (cAMP), which plays a critical role in the development of the beige phenotype and the activation of its thermogenic program in white adipose tissue (WAT). We investigated the metabolic effects of PDE3B inhibition with cilostazol treatment in the adipose tissue of high-fat diet (HFD)-fed mice. Seven-week-old male C57BL/6J mice were randomly assigned to either the cilostazol or control group. The control group was divided into two groups: the chow diet and HFD. The expression of uncoupling Protein 1 (UCP1) and other brown adipocyte markers was compared. In the HFD-fed cilostazol group, C57BL/6J mice displayed improvements in systemic metabolism, including improved glucose tolerance and lipid profile, but only modest effects on body weight were observed. In the visceral WAT of HFD-fed cilostazol-treated mice, cAMP/protein kinase A (PKA) signaling pathways were activated, resulting in the “browning” phenotype, smaller fat deposits, and enhanced mRNA expression of UCP1 and other brown adipocyte markers. PDE3B appears to be an important regulator of lipid metabolism, insulin sensitivity, and thermogenic programs in adipose tissues. An increase in intracellular cAMP via PDE3B inhibition with cilostazol treatment promoted the browning of visceral WAT.

## 1. Introduction

The prevalence of obesity is continuing to rise rapidly and is extensively associated with multiple comorbidities, such as cardiovascular diseases, diabetes, hypertension, and several cancers [1,2,3]. Adipose tissues, which consist of white (WAT) and brown adipose tissue (BAT), play an essential role in the regulation of whole-body energy homeostasis [4]. The excess expansion of WAT, due to a positive energy balance and defects in the thermogenic gene expression of BAT, is associated with obesity and various metabolic diseases [5]. It is well established that BAT plays a pivotal role in adaptive thermogenesis in rodents throughout their lifespan [6]. In human fetuses and newborns, BAT is found in axillary, cervical, perirenal, and periadrenal regions but decreases shortly after birth and has been considered to be irrelevant in adults. Recently, several investigations have shown that adults also have metabolically active BAT in the supraclavicular and paraspinal regions, which is activated when exposed to cold, and relatively high quantities of BAT are associated with lower body weight [7]. However, in humans, BAT is found in small volumes, with older and obese individuals often having negligible amounts [8].

Recent studies have revealed the presence of a subset of cells in WAT with very similar properties as BAT. These cells have been termed ‘beige’ adipocytes. Under basal conditions, beige adipocytes are similar to classic white adipocytes; however, they acquire a thermogenic signature similar to brown adipocytes in response to various stimuli such as cold exposure, hypercaloric diet, β-adrenergic stimulation, and exercise. This “beigeing” process has been suggested to have strong anti-obesity and antidiabetic effects in animals [9]. It has been shown that human pre-adipocytes from subcutaneous abdominal WAT can be differentiated into beige adipocytes upon stimulation with PPARγ agonist in vitro [10]. In addition, in humans, reduced insulin sensitivity is associated with the reduced expression of transcripts involved in BAT adipogenesis including MASK, MAP3K5, PPARγ, pRb, RXRγ, and PGC-1 in subcutaneous WAT [11]. When exposed to cold, there is an increase in catecholamine release, which then binds to the adrenergic receptor of BAT or beige adipose tissue and activates adenylyl cyclase to increase the intracellular concentration of cyclic adenosine monophosphate, which in turn activates the cascade of the protein kinase A (PKA)-dependent signaling pathway [12]. The cyclic adenosine monophosphate (cAMP)/PKA signaling pathway plays a critical in the development of the beige phenotype and the activation of its thermogenic program in WAT [12,13]. Therefore, it is important to maintain intracellular levels of cAMP during WAT browning [13].

Phosphodiesterase (PDE) plays a specific role in hydrolyzing cyclic guanosine monophosphate or cAMP and consists of 11 different protein groups [14]. There are two forms of PDE3: PDE3A and PDE3B. PDE3A and PDE3B exhibit unique and distinctive spatiotemporal patterns of expression: PDE3A is mainly located in platelets, trachea, and cardiovascular tissues, while PDE3B is located in organs involved in energy metabolism, such as the liver, pancreatic beta cells, and adipose tissues [15]. In a study with PDE3B knockout (KO) mice, it was observed that the epididymal WAT “browning” phenotype had smaller fat deposits and attenuated increases in body weight under a high-fat diet [16]. Cilostazol, a selective inhibitor of PDE3, was approved for the treatment of intermittent claudication and is known to elevate the intracellular level of cAMP by inhibiting its degradation [17]. Previous studies have also reported the pleotropic effects of cilostazol on metabolism including the modulation of glucose metabolism and inflammation [18,19,20,21,22]; however, none of the studies have investigated the browning effect of cilostazol including the expression of UCP1 and other brown adipocyte markers in WAT. Therefore, in this study, we hypothesized that the administration of cilostazol may induce WAT browning.

## 2. Materials and Methods

### 2.1. The Animal Study

Six-week-old male C57BL/6J mice were purchased from Jackson Laboratory (Bar Harbor, ME, USA). After 1-week acclimatization, the mice were randomly assigned to three groups depending upon the diet and drug treatment: group 1, chow fed for 16 weeks (*n*  =  5); group 2, high-fat diet (HFD; consisting of 60% fat, 20% carbohydrate, and 20% protein; total caloric energy = 5.24 kcal/g; D12492; Research Diet Inc., New Brunswick, NJ, USA) fed for 16 weeks (*n*  =  10); and group 3, HFD fed with cilostazol treatment (0.15% cilostazol) for 16 weeks (*n*  =  15). Throughout the experiment, the body weight and food intake were measured weekly. The mice were housed under standard conditions (21 ± 2 °C, 60 ± 10% humidity, 12 h light/dark cycle) with ad libitum access to food and water. All animal procedures were performed in accordance with the guidelines of the National Institutes of Health and pre-approved by the Animal Care and Use Committee of Yonsei University, College of Medicine (2017-0019; approved on 1 March 2017)

### 2.2. Oral Glucose Tolerance Test and Insulin Tolerance Test

The oral glucose tolerance test (OGTT) and insulin tolerance test (ITT) were performed after 16 weeks of diet administration. A glucose bolus of 1 g/kg body weight was administered orally after 6 h of fasting. After glucose administration, blood samples were collected via tail nick at 0, 15, 30, 60, and 120 min. The ITT was performed via the intraperitoneal injection of human regular insulin (5 units/kg body weight) (Sigma-Aldrich, Oakville, ON, Canada) into fasted mice. Blood samples were collected from the tail vein before administration and 15, 30, 60, and 120 min after insulin administration.

### 2.3. Biochemical Measurements

Blood glucose concentrations were measured using an Accu-Chek Performa glucometer (Boehringer-Mannheim, Indianapolis, IN, USA). As previously described [23], random blood glucose levels were measured from the tail blood of the random-fed (non-fasting) group using a glucometer. Serum insulin concentrations were determined using an enzyme-linked immunosorbent assay (Mouse Insulin ELISA Kit; Merck Millipore, Darmstadt, Germany). At the end of the treatment, the mice were euthanized and blood samples were collected via heart puncture. Serum levels of aspartate aminotransferase and alanine aminotransferase were measured using an enzyme-linked immunosorbent assay (ELISA; BioAssay Systems, Hayward, CA, USA). Serum triglyceride levels were measured using a triglyceride quantification kit (K622; BioVision, Milpitas, CA, USA). Hepatic triglyceride levels were determined using a triglyceride quantification kit after the homogenization of the liver tissue according to the manufacturer’s instructions.

### 2.4. Histologic and Immunohistochemical Analyses

Each AT was harvested and handled as described previously [24]. Briefly, three different adipose depots (subcutaneous WAT, visceral WAT and BAT) were identified and isolated; for BAT, the scapulae and corresponding depot was located and superficial white adipose atop the butterfly was removed and then the butterfly of interscapular brown fat was dissected. All AT were transferred to a 2 mL microcentrifuge tube and were frozen by immersion in liquid nitrogen and stored at −80 °C. The tissue samples (AT and liver) were washed, dehydrated, and embedded in paraffin. Some sections were stained with hematoxylin and eosin (H&E) to observe the histological structures. Sections of visceral and subcutaneous WAT and BAT were stained with an anti-UCP1 antibody (ab10983, Abcam, Cambridge, UK). Tissue samples were examined under a microscope and images were acquired using an attached digital camera. CellSens Entry software (Olympus, Tokyo, Japan) was used for image analysis. The adipocyte diameters were measured in 3–5 AT sections per mouse. The AT sections were stained with H&E and imaged at ×100 magnification prior to the characteristics being quantified using Adiposoft software 1.13 (National Institutes of Health, Bethesda, MD, USA) [25].

### 2.5. Cell Culture and Differentiation

3T3-L1 mouse preadipocyte cell lines were cultured as previously described [26]. Briefly, 2-day post-confluent cells (day 0) were cultured in Dulbecco’s modified Eagle’s medium (DMEM; Wel-GENE Inc., Daegu, Korea) supplemented with 10% fetal bovine serum (FBS; Wel-GENE Inc.), 2 μg mL^−1^ dexamethasone, 0.5 mM isobutyl-1-methylxanthine (Sigma-Aldrich), and 1 μg mL^−1^ insulin (Roche, Mannheim, Germany) for two days. The maturation medium, containing fresh DMEM supplemented with 10% FBS and 1 μg mL^−1^ insulin, was changed every two days. After seven days, fully differentiated adipocytes were harvested for the experiments. Harvested cells were treated with or without cilostazol, dibutyryl-cAMP(dbcAMP), or H89.

### 2.6. RNA Isolation and Real-Time Polymerase Chain Reaction Analysis

Total RNA was isolated from the cells using TRIzol reagent (Invitrogen, Carlsbad, CA, USA) following the manufacturer’s instructions. Complementary DNA (cDNA) was synthesized using the High-Capacity cDNA Reverse Transcription kit (Applied Biosystems, Foster City, CA, USA). The cDNA was then amplified in an ABI 7500 sequence detection system (Applied Biosystems) using Power SYBR Green PCR Master Mix (Applied Biosystems) under the following cycling conditions: 40 cycles of 95 °C for 5 s, 58 °C for 10 s, and 72 °C for 20 s. The primer sequences for mouse cDNA are described in Supplementary Table S1. The expression of the target genes was normalized to that of the reference gene glyceraldehyde-3-phosphate dehydrogenase (GAPDH). Quantitative analyses were conducted using the ΔΔcycle threshold method and StepOne Software version 2.2.2 (Applied Biosystems, Waltham, MA, USA).

### 2.7. Western Blot Analysis 

Western blotting was performed according to the standard protocol. Proteins were spectrophotometrically quantified using Bradford reagent. The tissue lysate was mixed with Laemmli buffer (2X, Bio-Rad, Hercules, CA, USA) supplemented with 5% β-mercaptoethanol in a 1:1 ratio. Proteins were separated using sodium dodecyl sulfate-polyacrylamide gel electrophoresis (SDS-PAGE) and transferred onto a polyvinylidene difluoride (PVDF) membrane via electrophoretic transfer (110 V for 1 h). Membranes were blocked and incubated with different antibodies, followed by incubation with secondary antibodies. Specific antibodies that target phospho-AMP-activated protein kinase (pAMPK; #2535S; Cell Signaling, Beverly, MA, USA) and GAPDH (#32233; Santa Cruz Biotechnology, Dallas, TX, USA) were used. The blots were visualized using enhanced chemiluminescence reagents (Amersham Pharmacia Uppsala, Sweden) according to the manufacturer’s protocol. The immunoreactive bands were quantified using ImageJ 4.1 software (NIH, Bethesda, MD, USA).

### 2.8. cAMP Direct Immunoassay

Cells were grown in 24-well plates. After treatment, cAMP levels were quantified by cAMP direct immunoassay kit (Bio Vision) following the manufacturer’s protocol.

### 2.9. Statistical Analysis

Data are expressed as the mean ± standard error (SE). Differences between groups were analyzed using one-way analysis of variance (ANOVA) with Bonferroni post hoc correction for multiple comparisons and two-way ANOVA with Bonferroni posttests for multiple variables. Statistical analyses were performed using SPSS (version 26.0; IBM Corp., Armonk, NY, USA) and graphs were plotted using GraphPad Prism (version 6.0; GraphPad, San Diego, CA, USA). Statistical significance was set at *p* < 0.05.

## 3. Results

### 3.1. Cilostazol Had Non-Significant Effects on Body Weight but Improved Glycemic Index in HFD Mice

**Figure 1:**
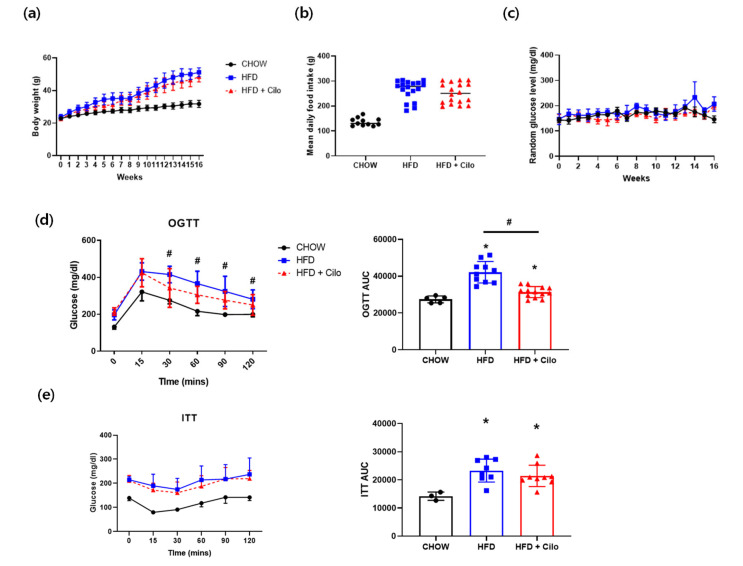
Cilostazol treatment had a modest effect on the bodyweight of mice with HFD-induced obesity and improved glycemic control. For 16 weeks, male C57BL/6J mice were fed with chow or HFD and treated with or without cilostazol. (**a**) Change in body weight; (**b**) average food intake per week; (**c**) changes in the mean random glucose levels during the 16-week experiment; and (**d**) oral glucose tolerance test and differences in the associated AUC; (**e**) insulin tolerance test and differences in the associated AUC. Data are presented as the mean ± standard error. * *p* < 0.05 vs. corresponding chow control value and # *p* < 0.05 vs. corresponding HFD control value. HFD, high-fat diet; Cilo, cilostazol; OGTT, oral glucose tolerance test; ITT, insulin tolerance test; AUC, area under the curve.

**Figure 2:**
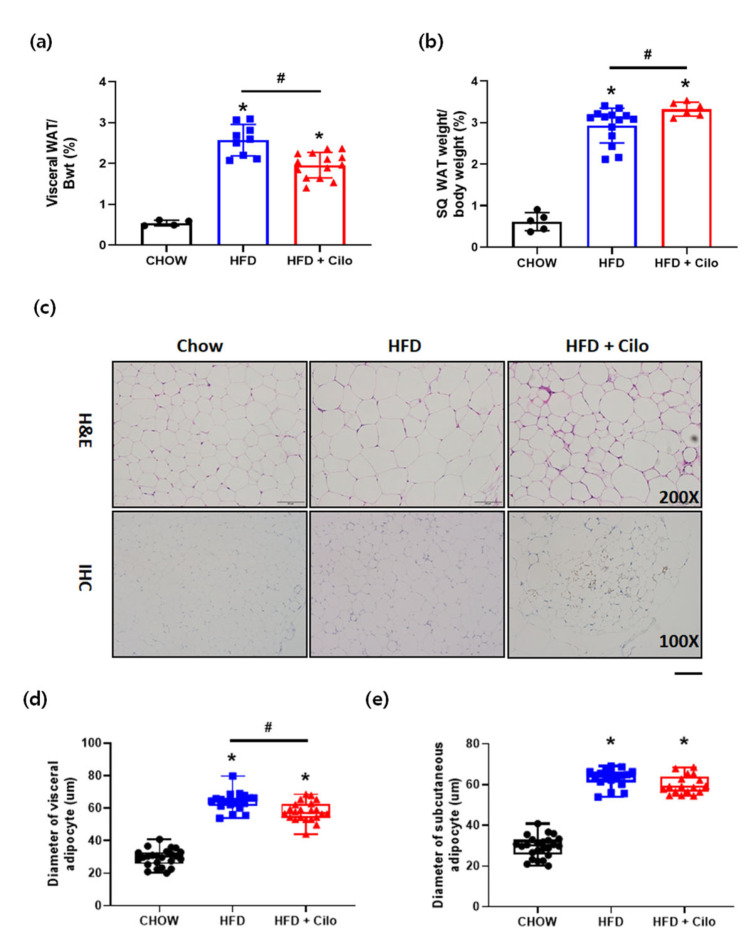
Cilostazol treatment modified WAT and reduced the size of adipocytes in visceral WAT. (**a**) Changes in visceral fat weight; (**b**) changes in subcutaneous fat weight; (**c**) representative histological images of H&E-stained visceral and subcutaneous WAT (×200 magnification) of chow control, HFD control, and cilostazol-treated HFD mice; (**d**) diameters of visceral adipocyte tissue from chow control, HFD control, and cilostazol-treated HFD mice; and (**e**) diameters of subcutaneous adipocyte tissue from chow control, HFD control, and cilostazol-treated HFD mice. * *p* < 0.05 vs. corresponding chow control value and # *p* < 0.05 vs. corresponding HFD control value. HFD, high-fat diet; Cilo, cilostazol.

Given the potential clinical relevance of cAMP for understanding the mechanisms underlying the induction of browning in WAT depots, 7-week old male mice were fed a HFD with or without cilostazol to prompt diet-induced obesity. The body weight of both the HFD control and cilostazol-treated HFD mice was significantly greater than that of the chow control mice (Figure 1a). Cilostazol treatment only had a modest effect on the overall body weight (Figure 1a), with no statistical difference in the HFD groups. In addition, the daily food intake and random glucose levels did not differ over the HFD groups (Figure 1b,c). In the OGTT, compared with the HFD control group, the cilostazol-treated HFD group exhibited a significant improvement in glucose tolerance at 30, 60, 90, and 120 min after glucose administration (Figure 1d). The AUC of the OGTT was significantly lower in the cilostazol-treated HFD group than in the HFD control group (Figure 2a). However, for the ITT, the cilostazol group displayed a non-significant improvement in glucose levels compared to the HFD control group (Figure 1e). The difference in the AUC values of the ITT was marginally significant (*p* = 0.051) between the HFD groups (control vs. cilostazol-treated) (Figure 1e).

### 3.2. Cilostazol Reduced Visceral WAT and the Size of the Visceral Adipocytes Isolated from HFD Mice

We investigated whether cilostazol has a beneficial effect on WAT remodeling. Compared to HFD control mice, the percentage of visceral WAT weight relative to body weight was significantly lower in cilostazol-treated HFD mice (Figure 2a), whereas the percentage of subcutaneous WAT weight relative to body weight was significantly higher (Figure 2b). Moreover, cilostazol administration markedly reduced the size of the visceral adipocytes, as evident in the histological analyses (Figure 2c). The average diameter of the visceral adipocytes was significantly decreased, suggesting that cilostazol ameliorated the adipocyte hypertrophy caused by the HFD, resulting in smaller adipocytes (Figure 2d), but the size was still bigger than that in chow control mice. Regarding subcutaneous WAT, there was a non-significant reduction (Figure 2e).

We then examined the mRNA expression of genes related to chronic inflammation in visceral fat tissue (Supplementary Figure S1). The expression levels of proinflammatory CD11c^+^ adipose tissue macrophage were significantly higher in HFD control mice than in chow control mice. Treatment with cilostazol decreased the mRNA expression of CD11c in HFD mice.

### 3.3. Cilostazol Increased Brown Adipocyte Maker Gene Expressions in the Visceral WAT Isolated from HFD Mice

**Figure 3:**
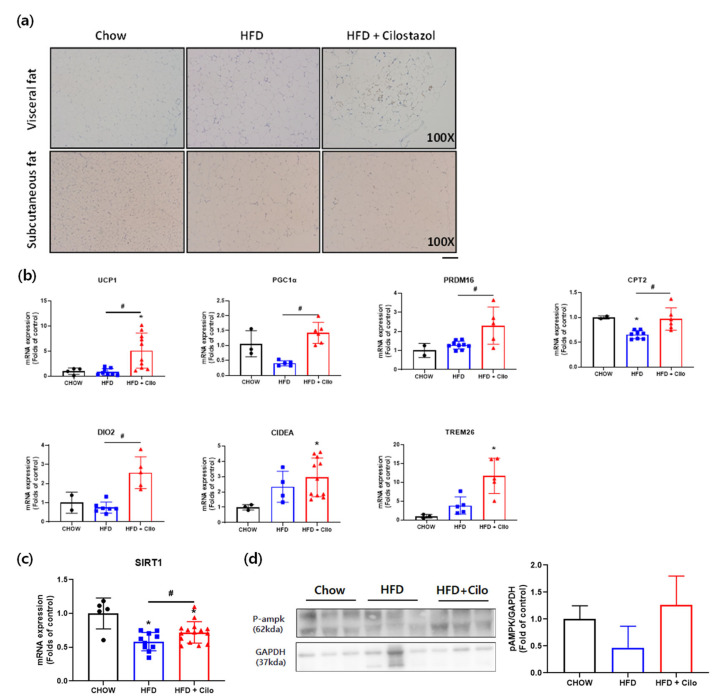
Cilostazol treatment-induced white-to-beige phenotypic conversion of WAT in HFD mice. (**a**) Representative histological images of UCP1 immunohistochemical-stained (brown) visceral and subcutaneous WAT (×100 magnification) of chow control, HFD control, and cilostazol-treated HFD mice; (**b**) RT-PCR analysis of browning genes in visceral WAT; (**c**) relative mRNA expression levels of thermogenesis-related genes and sirtuin 1 (SIRT1) in visceral fat; and (**d**) protein levels of phosphorylated AMP-activated protein kinase (pAMPK) in visceral fat were determined using Western blot analysis. The graph on the right shows the densitometric analysis of the pAMPK/glyceraldehyde 3-phosphate dehydrogenase (GAPDH) ratio determined from the immunoblots shown on the left. * *p* < 0.05 vs. corresponding chow control value and # *p* < 0.05 vs. corresponding HFD control value. HFD, high-fat diet; Cilo, cilostazol.

To address whether cilostazol promoted WAT browning, we performed uncoupling protein 1 (UCP1) immunohistochemistry staining, which demonstrated enhanced UCP1 signals in the visceral WAT of cilostazol-treated HFD mice, whereas no differences were noted in subcutaneous WAT (Figure 3a). The relative mRNA levels of UCP1 and other thermogenesis-related genes, including PGC-1α, PRDM16, CPT2, DIO2, CIDEA and TMEM26 [27] were also significantly upregulated in visceral WAT by cilostazol treatment (Figure 3b) than the HFD control mice but no significant differences were noted between the chow control mice and cilostazol-treated HFD mice. In addition, the mRNA levels of Sirt1 were significantly increased in visceral fat by cilostazol treatment (Figure 3c). The decreased phosphorylation of AMPK in the visceral fat of the HFD control mice was recovered by cilostazol treatment (Figure 3d). These data suggest that cilostazol induces brown fat-like changes via the activation of the AMPK/SIRT1 pathway.

### 3.4. Cilostazol Increased Brown Adipocyte Marker Gene Expressions in 3T3-L1 Adipocytes

**Figure 4:**
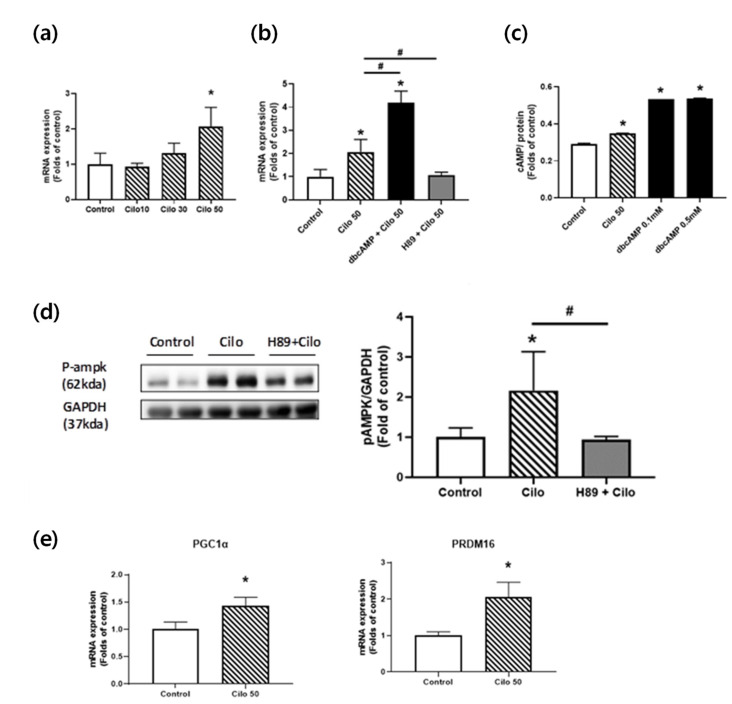
Cilostazol treatment-induced white-to-beige phenotypic conversion in 3T3-L1 cells. (**a**) RT–PCR analysis of vehicle- or cilostazol-treated 3T3-L1 cells for UCP1 transcripts (*n* = 5 experiments); (**b**) RT–PCR analysis of UCP1 in 3T3-L1 adipocytes after cilostazol treatment with dbcAMP (0.1 nM) or PKA inhibition by H89 (10 μM); (**c**) cAMP levels in vehicle-, cilostazol-, and dbcAMP-treated 3T3-L1 cells; (**d**) protein levels phosphorylated AMP-activated protein kinase (pAMPK) in 3T3-L1 cells were determined using Western blot analysis. The graph on the right shows the densitometric analysis of the pAMPK/glyceraldehyde-3-phosphate dehydrogenase (GAPDH) ratio determined from the immunoblots shown on the left. Left, vehicle-treated 3T3-L1 adipocytes. Middle, cilostazol-treated 3T3-L1 adipocytes. Right, cilostazol- and H89-treated 3T3-L1 adipocytes; and (**e**) RT–PCR analysis of vehicle- or cilostazol-treated 3T3-L1 cells for PGC-1α and PRDM16 transcripts (*n* = 5 experiments). * *p* < 0.05 vs. corresponding control value and # *p* < 0.05 vs. corresponding cilostazol-treated 3T3-L1 adipocytes value. Cilo, cilostazol.

In an in vitro study using 3T3-L1 adipocytes, cilostazol significantly increased UCP1 mRNA levels in a dose-dependent manner (Figure 4a). The incubation of differentiated 3T3-L1 adipocytes with cilostazol also amplified the expression of UCP1 induced by dbcAMP, a cAMP analog. Then, we utilized H89, a PKA inhibitor to examine the regulatory role for cAMP and PKA in the induction of UCP1 in cilostazol-treated 3T3-L1 adipocytes. The induction of UCP1 by cilostazol was inhibited by H89, suggesting that cAMP and PKA have a regulatory role (Figure 4d). We then examined whether the UCP1 expression was increased by cAMP-mediated pathways. The incubation of differentiated 3T3-L1 adipocytes with cilostazol or dbcAMP increased the intracellular cAMP levels (Figure 4c). As shown in Figure 4c,d, cilostazol increased intracellular cAMP levels and AMPK phosphorylation, demonstrating the role of PDE3B in the regulation of signaling pathway(s) that activates AMPK through alterations in cAMP levels. Then, we investigated whether AMPK signaling is responsible for the upregulation of PGC1α and the induction of PRDM16. PGC1α and PRDM16 expression was significantly increased in differentiated 3T3-L1 adipocytes treated with cilostazol.

### 3.5. Cilostazol Attenuated HFD-Induced Accumulation of Large Lipid Droplets in BAT- and HFD-Induced Impairment of BAT Activity

**Figure 5:**
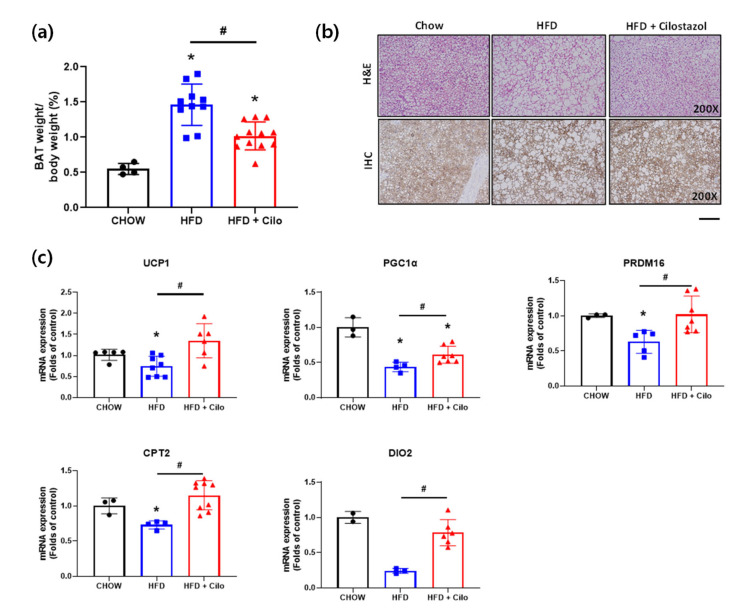
Cilostazol enhanced expressions of brown thermogenic transcriptional factors in brown fat. (**a**) Brown fat weight; (**b**) representative histological images of hematoxylin and eosin (H&E) stained and UCP1 immunohistochemical (IHC) stained (brown) brown adipose tissue section (magnification, ×200, respectively) of Chow control, HFD control and cilostazol-treated HFD mice, respectively; (**c**) relative mRNA expression levels of thermogenesis-related genes in the brown fat. * *p* < 0.05 vs. corresponding chow control value and # *p* < 0.05 vs. corresponding HFD control value. HFD, high fat diet; Cilo, cilostazol.

To determine whether cilostazol affected BAT, interscapular BAT was examined. While the overall amount of BAT was significantly higher in both HFD groups than the chow control group (Figure 5a), cilostazol attenuated the HFD-induced accumulation of unilocular large lipid droplets within brown adipocytes. In other words, multilocular lipid droplets identified by H&E staining and UCP1-positive appearance were comparable between the cilostazol-treated HFD and HFD control groups (Figure 5b). Moreover, cilostazol significantly attenuated the HFD-induced impairment of BAT activity, as assessed by the mRNA expression of UCP1, PGC1α, PRDM16, CPT2, and DIO2, which are key transcriptional regulators of thermogenesis in BAT (Figure 5c). There was no significant difference in BAT activity between the chow control mice and cilostazol-treated HFD mice.

### 3.6. Cilostazol Attenuated HFD-Induced Hepatic Steatosis

**Figure 6:**
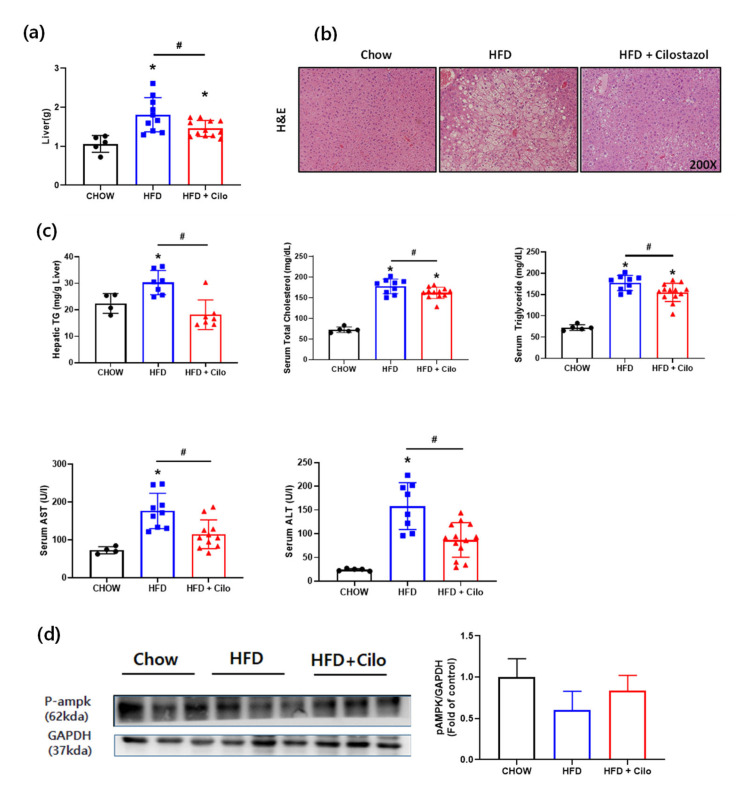
Cilostazol-attenuated HFD-induced hepatic steatosis. (**a**) Liver weight; (**b**) hematoxylin and eosin (H&E) staining of liver sections. Magnification, ×200; (**c**) hepatic TG concentration, serum total cholesterol, triglyceride, aspartate aminotransferase (AST), and alanine aminotransferase (ALT) levels; (**d**) protein levels of phosphorylated AMP-activated protein kinase (pAMPK) in the liver were determined using Western blot analysis. The graph on the right shows the densitometric analysis of the pAMPK/glyceraldehyde 3-phosphate dehydrogenase (GAPDH) ratio determined from the immunoblots shown on the left. * *p* < 0.05 vs. corresponding chow control value and # *p* < 0.05 vs. corresponding HFD control value. HFD, high fat diet; Cilo, cilostazol.

Observations of the livers of the mice showed that cilostazol treatment attenuated the HFD-induced accumulation of lipids in the liver. Cilostazol treatment for 16 weeks significantly attenuated the HFD-induced increase in liver weight (Figure 6a). Liver histology by H&E staining showed that cilostazol treatment markedly attenuated the HFD-induced aberrant lipid accumulation (Figure 6b). While plasma total cholesterol (TC), triglyceride (TG), aspartate aminotransferase (AST), and alanine aminotransferase (ALT) levels were increased in HFD mice compared to chow mice, cilostazol treatment attenuated these HFD-induced increases (Figure 6c). Moreover, cilostazol reduced the hepatic TG content, which was comparable to that in the control HFD mice. As AMPK plays a crucial role in hepatic steatosis [28], we then examined whether cilostazol ameliorated lipid accumulation via AMPK. The expression levels of AMPK phosphorylation were significantly reduced in HFD control mice than chow control mice (Figure 6d). The decreased phosphorylation of AMPK in the liver of the HFD control mice was recovered by cilostazol treatment, indicating the role of PDE3B in the regulation of signaling pathway(s) that activates AMPK in liver.

## 4. Discussion

White adipose browning, a process that transforms energy-storing white adipocytes into heat-producing beige adipocytes, is an attractive strategy for increasing energy expenditure and treating obesity and diabetes. Therefore, discovering compounds with WAT browning effects is of great importance for providing alternative approaches for the treatment of these common but serious conditions. Previous studies with PDE3B KO mice demonstrated the characteristics of a beige phenotype, with a smaller increase in body weight under HFD, smaller fat deposits, an increased expression of genes related to the recruitment of beige adipocytes, increased UCP1, and increased fatty acid oxidation, and increased O_2_ consumption and energy dissipation [16]. On the other hand, in a study with mice overexpressing PDE3B, there was an early and striking appearance of hyperglycemia, islet dysfunction, glucose intolerance, insulin resistance, and hepatic steatosis when challenged with HFD [29]. In this study, we present clear data supporting the function of PDE3B inhibition in raising energy expenditure by promoting WAT browning and highlight the possible therapeutic effects of cilostazol on obesity and obesity-related complications, such as hepatic steatosis.

In this study, cilostazol treatment resulted in an improved glycemic index, including significant reductions in glucose tolerance and marginally significant improvements in insulin sensitivity. Cilostazol treatment significantly decreased the size of fat pads and adipocytes in visceral WAT while the whole body and subcutaneous WAT content was increased, suggesting the association between cilostazol treatment and WAT remodeling. In previous studies with 3T3-L1 preadipocytes, cilostazol increased the transcriptional activity of PPARγ, of which activation is associated with fat redistribution [30,31,32]. Taken together with previous studies, we can speculate that cilostazol, through PPARγ, is associated with adipose tissue remodeling and redistribution. Moreover, cilostazol treatment resulted in the activation of several genes crucial for BAT, such as PRDM16, PGC1α, DIO2, and CPT2, in the visceral WAT, interscapular BAT of HFD mice, and 3T3L1 adipocytes. However, changes in body weight were modest, suggesting that additional stimuli may be required to induce more functional brown adipocyte differentiation.

Cilostazol is an antiplatelet drug that inhibits both primary and secondary platelet aggregation in response to ADP, collagen, epinephrine, and arachidonic acid [17]. The antiplatelet, anti-inflammatory, and vasodilator actions of cilostazol improve the claudication intermittent symptoms via the cAMP-induced inhibition of PDE [33,34,35]. Furthermore, there is evidence to support the role of PDE inhibition in improving systemic metabolism. Studies have demonstrated a beneficial effect of cilostazol on lipoprotein metabolism, characterized by an increase in high-density lipoprotein cholesterol and a reduction in plasma triglyceride levels via cAMP-induced increase in lipoprotein lipase activity [36,37,38]. Moreover, cilostazol treatment prompted improvements in insulin resistance in diabetic animal models [18,19,20,21] and ameliorated hepatic steatosis as well as autoimmune hepatitis [22,39]. However, the pharmacological efficacy of cilostazol in obesity has not yet been investigated.

cAMP is important for the development of the beige phenotype and the activation of its thermogenic program [40]. As demonstrated in this study, the inhibition of PDE3B by cilostazol treatment increased the intracellular concentration of cAMP with subsequent AMPK signaling, the integration of which resulted in the upregulation of PGC1α and the induction of PRDM16. In other words, in the visceral WAT of cilostazol-treated HFD mice, PRDM16 initiated a coordinated metabolic program by upregulating PGC-1α, DIO2, and critical mitochondrial proteins, including UCP1 and CPT2, which might be responsible for increased thermogenesis. In 3T3-L1 adipocytes, the downregulation of PDE3B via pharmacological inhibition with cilostazol also increased the intracellular concentration of cAMP with subsequent AMPK signaling, resulting in the activation of PGC-1α and the enhanced induction of UCP1 mRNA. Thus, we suggest that PDE3B may regulate a cAMP-sensitive molecular “switch” for the browning of visceral WAT.

Our data support the notion that increased cAMP signaling in cilostazol-treated mice leads to the activation of AMPK signaling. Studies of 3T3L1 adipocytes have indicated that cilostazol increases AMPK phosphorylation, suggesting that PDE3B regulates a cAMP “pool” that is important in the regulation of AMPK. AMPK is a key regulator of energy metabolism and mitochondrial biogenesis [41]. The promotive effects of AMPK on WAT browning and BAT activity have been extensively investigated [41]. It has been reported that the modulatory functions of myostatin, adiponectin, and irisin on WAT browning involve AMPK activation [42,43,44]. Adiponectin, a circulating WAT-specific adipocytokine, was significantly increased in PDE3B KO mice [45]. Similarly, the mRNA expression of adiponectin was significantly decreased in HFD mice than in chow control mice while cilostazol treatment increased adiponectin mRNA expressions in HFD mice in this study (Supplementary Figure S2). Although the effect of cilostazol on adiponectin secretion in visceral WAT has not been studied, it is known that white adipocyte adiponectin exocytosis is triggered by cAMP and a concomitant increase in cytosolic Ca^2+^ potentiates its release, suggesting the role of PDE3b inhibition in the secretion of adiponectin [46]. Experiments with 3T3L1 adipocytes have shown that the browning-promotive effects of cilostazol are substantially abolished by treatment with H89, a PKA inhibitor, suggesting a regulatory role on cAMP, PKA, and AMPK signaling. Although further investigations, such as animal experiments using systemic or adipose-specific AMPK KO mice, are required to prove that AMPK is the cause of fat browning in vivo, the current data suggest that AMPK activation may involve cilostazol-induced WAT browning, at least in vitro.

Beige and brown adipocytes are recognized as having a potential role in the defense against obesity by dissipating energy. Variations in the activation and amount of BAT may regulate energy expenditure, but BAT is inversely correlated with body weight and decreases with age [6]. In this study, the amount of interscapular BAT in cilostazol-treated HFD mice was lower than that in control HFD mice. However, the level of UCP1 mRNA and morphological assessment of numerous unilocular lipid droplets were comparable between the interscapular BAT from HFD control and cilostazol-treated mice. Cilostazol attenuated HFD-induced lipid accumulation in BAT and impaired BAT activity. In fact, cilostazol increased the intracellular concentration of cAMP in brown adipocytes, which stimulated the expression of thermogenic and brown-specific genes, thus enhancing metabolic activity.

WAT is divided into two main types, visceral and subcutaneous, which differ regarding gene expression, metabolic profile, and cell composition [47]. Visceral WAT is more resistant to acquiring a thermogenic profile than subcutaneous WAT [48]. However, in this study, the effect of cilostazol was only apparent in visceral WAT, whereas minimal changes were noted in subcutaneous WAT. Although further studies are warranted to elucidate these findings, speculations can be made based on a study concerning the alteration in the activity and expression of cyclic nucleotide PDE families in omental and subcutaneous adipose tissue and adipocytes in obese humans [49]. There were inverse correlations between the body mass index and PDE3 activities in the adipocytes isolated from omental WAT, while no correlation was found in the subcutaneous WAT of the obese subjects [49].

This study has several limitations. First, the administration of cilostazol may not be reliable in all animals. Cilostazol injection into the peritoneal cavity may have been a more appropriate technique in this setting; however, intraperitoneal delivery may induce painful ileus and peritonitis in rodents, with subsequent adhesions [50]. Second, the size of the study population was not sufficient for several analyses. Therefore, we cannot exclude the possibility that the statistically insignificant results may still have clinical significance. Third, although we provided evidence for the amelioration of hepatic steatosis with cilostazol treatment, we were not able to elucidate the underlying mechanism, which requires further investigation.

## 5. Conclusions

Cilostazol promoted the beigeing of visceral WAT, increased BAT activity, and improved the glycemic index and hepatic steatosis. Overall, our data indicate that cilostazol exerts multiple beneficial effects on both white and brown adipocytes, and may possess therapeutic potential for obese patients with diabetes.

## Data Availability

Not applicable.

## Supplementary Materials

The following supporting information can be downloaded at: https://www.mdpi.com/article/10.3390/biomedicines10081852/s1, Supplementary Figure S1: Effects of cilostazol administration on mRNA expression of CD11c in the visceral WAT; Supplementary Figure S2: Effects of cilostazol administration on mRNA expression of adiponectin in the visceral WAT.
