# Supplementary material for: Effects of a Phosphodiesterase inhibitor on the Browning of Adipose Tissue in Mice
Source: Biomedicines. 2022 Aug 1;10(8):1852. doi: 10.3390/biomedicines10081852 (PMC9405663; doi:10.3390/biomedicines10081852)
Supplement: Supplementary file 1 [file biomedicines-10-01852-s001.zip › biomedicines-1764068-supplementary.pptx]

## Slide 1
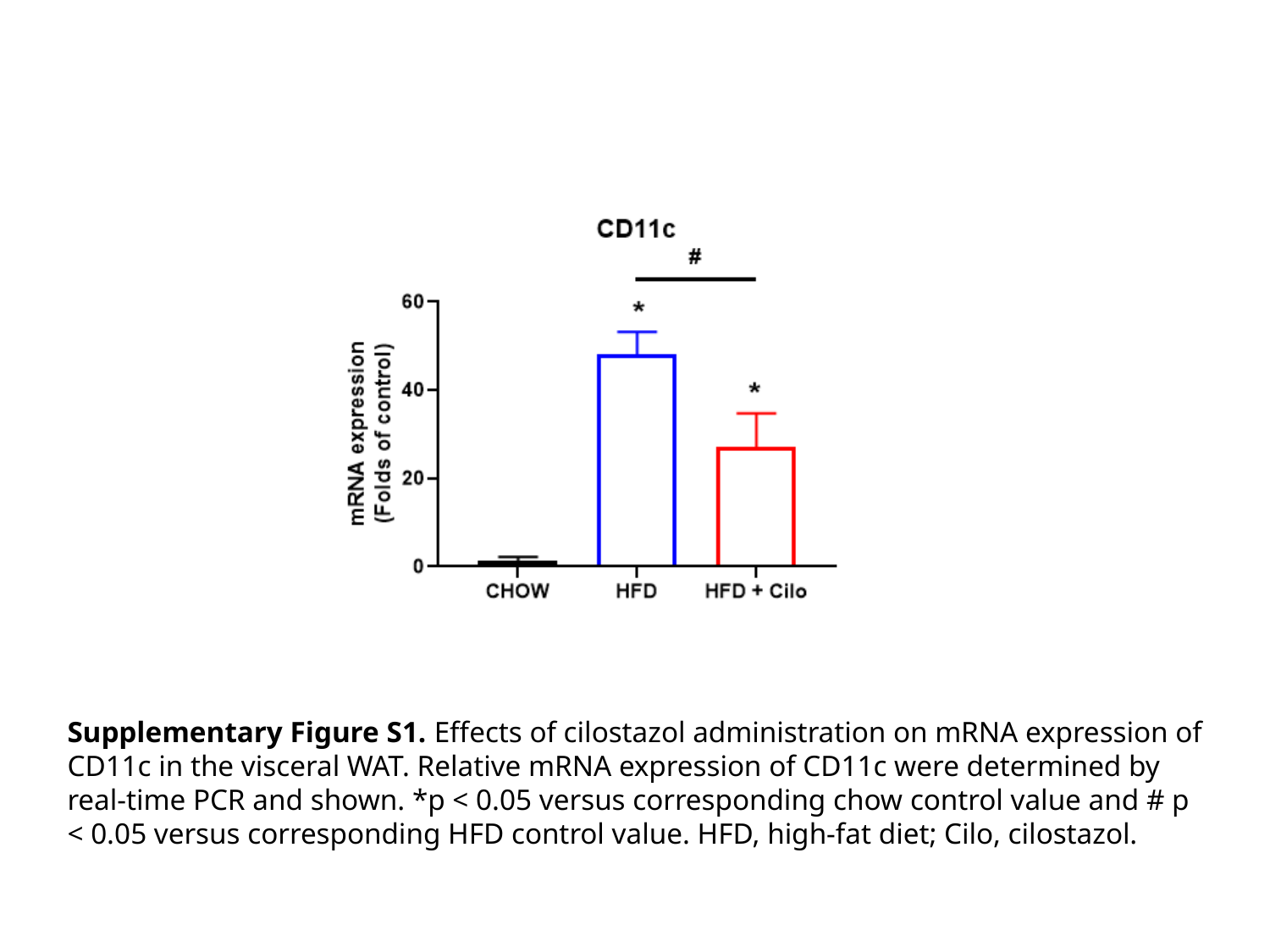

Supplementary Figure S1. Effects of cilostazol administration on mRNA expression of CD11c in the visceral WAT. Relative mRNA expression of CD11c were determined by real-time PCR and shown. *p < 0.05 versus corresponding chow control value and # p < 0.05 versus corresponding HFD control value. HFD, high-fat diet; Cilo, cilostazol.

## Slide 2
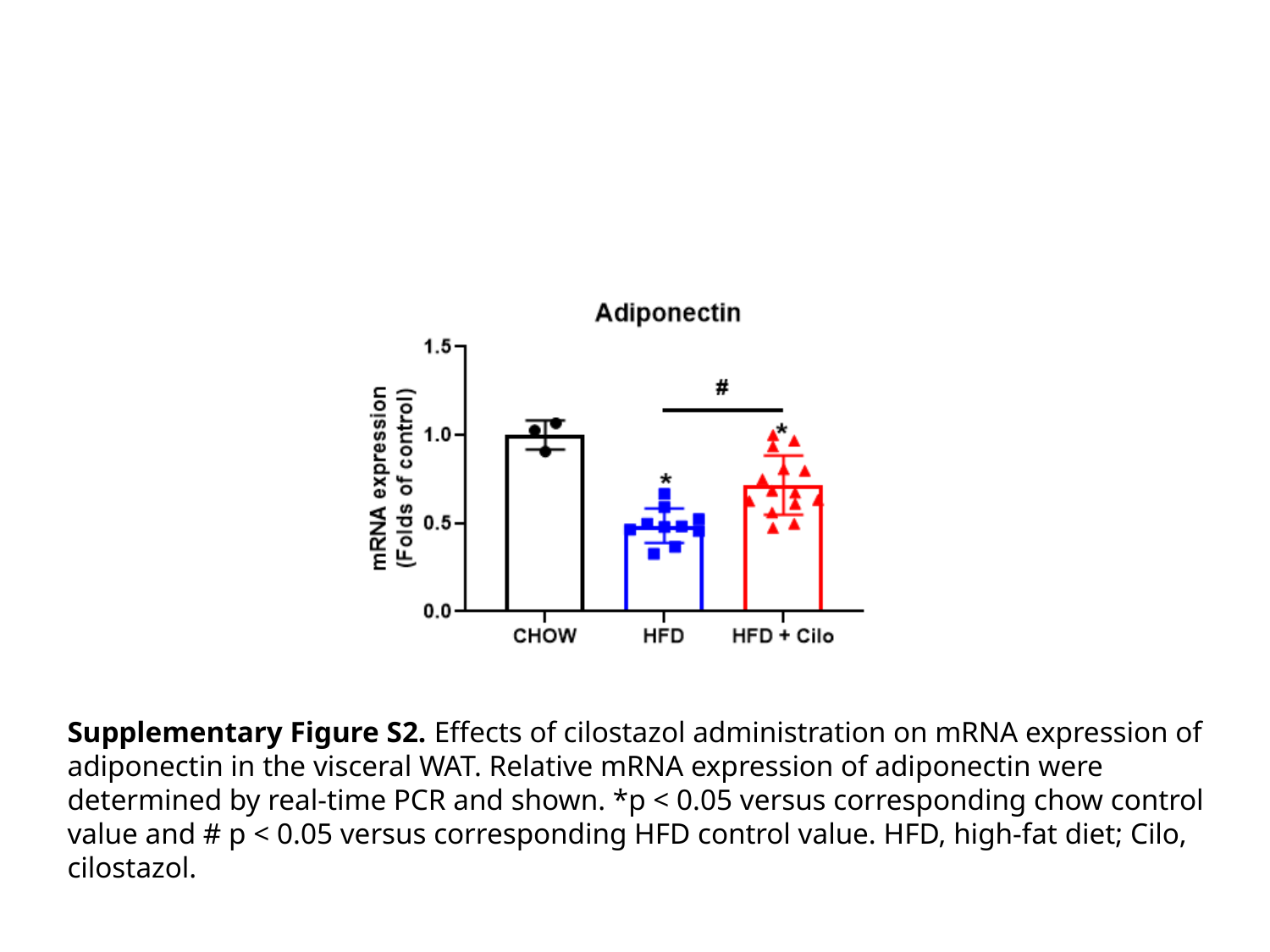

Supplementary Figure S2. Effects of cilostazol administration on mRNA expression of adiponectin in the visceral WAT. Relative mRNA expression of adiponectin were determined by real-time PCR and shown. *p < 0.05 versus corresponding chow control value and # p < 0.05 versus corresponding HFD control value. HFD, high-fat diet; Cilo, cilostazol.
